# “You can’t live for yourself, can you? That’s just boring”: perspectives on social health as a relational concept as described by community-dwelling older adults in a large Dutch city

**DOI:** 10.1093/geront/gnag032

**Published:** 2026-03-23

**Authors:** Isabelle F van der Velpen, Eline Verspoor, Mohammad Arfan Ikram, Meike W Vernooij, René J F Melis, Myrra J F Vernooij-Dassen, Marieke Perry

**Affiliations:** Department of Epidemiology, Erasmus MC, Rotterdam, The Netherlands; Department of Radiology and Nuclear Medicine, Erasmus MC, Rotterdam, The Netherlands; Department of Geriatric Medicine, Radboudumc Alzheimer Center, Radboud University Medical Center, Nijmegen, The Netherlands; Department of Epidemiology, Erasmus MC, Rotterdam, The Netherlands; Department of Epidemiology, Erasmus MC, Rotterdam, The Netherlands; Department of Radiology and Nuclear Medicine, Erasmus MC, Rotterdam, The Netherlands; Department of Geriatric Medicine, Radboudumc Alzheimer Center, Radboud University Medical Center, Nijmegen, The Netherlands; Radboud Institute for Health Sciences, Radboud University Medical Center, Nijmegen, The Netherlands; Department of IQ Healthcare, Radboud University Medical Center, Nijmegen, The Netherlands; Department of Geriatric Medicine, Radboudumc Alzheimer Center, Radboud University Medical Center, Nijmegen, The Netherlands; Department of Primary and Community Care, Radboud University Medical Center, Nijmegen, The Netherlands

**Keywords:** Qualitative research, Social relationships, Core ties, Reciprocity, Social health definition

## Abstract

**Background and Objectives:**

Social health is an essential domain of health, but the perspective of older adults themselves on the concept is lacking. We aimed to qualitatively explore older adults’ perspectives on the concept of social health and how it relates to overall well-being and health during aging.

**Research Design and Methods:**

Seventeen out of 35 invited participants of the Rotterdam Study aged 60–74 took part in semistructured in-depth interviews. We used a purposive sampling strategy while safeguarding age, sex, and ethnic diversity. A thematic analysis on the interview transcripts was performed.

**Results:**

Five themes were identified that capture older adults’ perspective on social health. First, autonomy and meaning are important conditions for social health, and are influenced by identity. Second, social health is shaped in interaction with the immediate social environment, where reciprocated relationships with close others are valued. Third, individuals interact with the broader societal environment, which relates to social participation within their needs and interpretations of social norms. Fourth, physical and mental health of individuals and their loved ones are preconditions for stable social health with aging. Finally, core relationships were central to all other themes and mainly involve close family ties.

**Discussion and Implications:**

According to older adults, social health is established in multiple domains of social interaction. Reciprocated social interaction with core ties is considered a critical component of social health. In promoting social health, care should be taken to honor/integrate older adults’ perspective on social health as a relational rather than an individual concept.

## Background and objectives

**Figure 1:**
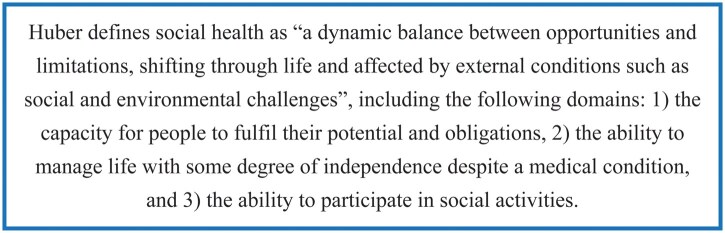
Definition of social health by Huber et al. (2011).

As populations worldwide age, comprehensive health promotion tailored to older adults should be a major clinical and public health goal. Social health is one of the three domains of health according to the World Health Organization’s (WHO) definition and its adaptation by Huber et al. (2011) (Figure 1). Whereas the WHO defines health as “a state of complete physical, mental and social well-being and not merely the absence of disease or infirmity,” social health in Huber’s definition takes a positive approach to health, focusing on remaining capacities and abilities in the presence of a medical condition. This is specifically relevant in the context of aging and increasing (multi)morbidity. The concept has since been further developed, recently leading to a new definition of social health as “a reciprocal relational concept in which well-being is defined by how an individual relates to their social environment and how the social environment relates to the individual” (Vernooij-Dassen et al., 2022). Social health is an umbrella concept that captures many different aspects of social relationships and interaction, including but not limited to social network structure and function, social support, social engagement, autonomy, dignity, and loneliness. A distinguishing feature of social health as a concept is that it is part of health, rather than an entity outside of health.

Several frameworks have aimed to capture how social interaction is organized and how it may affect health outcomes. Berkman et al. developed a framework on how macrolevels, mezzolevels, and microlevels of social relationships affect health through psychological, health-behavioral, and physiological pathways (Berkman et al., 2000). Holt-Lunstad provided a systems approach that identifies how social connections may causally affect physical health outcomes, in which four dimensions of social networks were proposed: individual level, family and close relationships, community, and society (Holt-Lunstad, 2018). Crucially, these frameworks position social relationships as a separate entity outside of health, rather than as a part of overall health. Considering social health as part of health facilitates the recognition of the interconnection of social health with mental and physical health, which may be more recognized by health policy and management.

Many qualitative studies revealed the importance of social aspects to older persons’ overall health and well-being, quality of life, and successful aging. Social relationships, activities, and other social components take a central role in older adults’ perspectives on and experiences with well-being and health (Asghar et al., 2019; Elo et al., 2011; Idler et al., 1999; Kirkby-Geddes & Macaskill, 2016; Tkatch et al., 2017), as well as in studies on frailty (Puts et al., 2009) and healthy aging in place (Bosch-Farre et al., 2020; Leung, 2022; Molina-Mula et al., 2020). Social relationships have further been found to be related to dignity, resilience, quality of life, joy of life and decision making in older age (Doekhie et al., 2020; Domajnko & Pahor, 2015; Imanzadeh & Hamrahzdeh, 2018; Rinnan et al., 2018; Tabari et al., 2016). Social factors arose as essential parts of community-based intervention programs when older adults were asked for their take on such programs: peer support in the program, addressing social networks and social cohesion were seen as strengths and motivators in programs geared toward mild frailty or health promotion (Frost et al., 2018; Hilger-Kolb et al., 2019; Lette et al., 2015; Yu et al., 2020). Social support was even a prerequisite for success in a qualitative study on a sarcopenia intervention program (von Berens et al., 2018). This body of qualitative literature suggests that, to older adults, social relationships are part of health. This body of existing literature on various subdomains of social health is very important, but the perspective of older adults themselves on the concept of social health is still lacking. Insight into their ideas is crucial to be able to help them improve their social health and ultimately their quality of life.

In this study, we aimed to explore the perspectives of older adults on the concept of social health, especially on how older adults experience the interaction between the social environment and their own social functioning, and the relation to their overall health.

## Research design and methods

### Participants and setting

We applied a qualitative design using in-depth, semistructured interviews. This study took place within the Rotterdam Study, a prospective population-based cohort study that started in 1990 in the Ommoord neighborhood in Rotterdam (Ikram et al., 2024). Inhabitants aged ≥40 years are invited to participate in the Rotterdam Study and are followed-up every 3–4 years. The Rotterdam Study has been approved by the Medical Ethics Committee of the Erasmus MC (registration number MEC 02.1015) and by the Dutch Ministry of Health, Welfare and Sport (Population Screening Act WBO, license number 1071272-159521-PG). All participants provided written informed consent to participate in the study. Participants from the third subcohort of the Rotterdam Study were invited for the current interview study. This subcohort was selected based on participants’ age (range: 59–97 years). Extensive recent sociodemographic information was available for 2513 participants, based on questionnaires that were sent out to all living Rotterdam Study participants from April to August 2020 (Licher et al., 2021). This information was combined with amnestic and genetic ancestry data to obtain an estimate of participant ethnicity. A purposive sampling strategy was applied, based on age (<65/>65 years), sex (male/female), ethnicity (native Dutch/Surinamese/Indonesian/Dutch Caribbean/Other), employment status (employed/retired), education level (primary education/lower intermediate/higher intermediate/higher education), marital status (married/widowed/divorced/single), and presence of feelings of loneliness (yes/no) on a questionnaire during the COVID-19 pandemic (van der Velpen et al., 2024). A sample of 35 participants was purposively selected through this strategy to be invited for the study. Participants received an interview study information letter by regular mail and were contacted by telephone one week later to be invited. Information letters were sent from May 2021 to September 2021. In total, 17 participants agreed to be interviewed. Of the remaining 18 eligible participants, 12 refused to participate, and another 6 did not respond to the telephone invitation before the end of data collection (November 2021). Those who refused to participate gave as reasons that they were not interested or did not currently have time. Refusers did not differ from participants on sampling characteristics. Nonresponders were more often female and more often employed compared to other participants and refusers. There was no drop-out among participants who agreed to do the interview. Interviews took place in the participant’s home or in the Rotterdam Study research center, based on the participant’s preference. During 3 interviews (#3, #14, #16), the participant’s partner was present during (parts of) the interview. For all other interviews, no nonparticipants were present. Written informed consent was provided by all participants before start of the interview.

### Data collection

Interviews were conducted from June to November 2021. Due to the ongoing COVID-19 pandemic at that time, interviews were conducted within a short time period to stay ahead of new lockdown measures. All interviews were conducted by the same researcher (IV). Researcher characteristics are described below. Interviews were semistructured. The researcher used an interview guide with topics and prompts for guidance. The interview guide was pilot-tested and was not shared with participants beforehand. During data collection, the interview guide was slightly adapted after interview #12 to change one topic from “dementia prevention programs” to “expectations on personal social health in the near future,” since questions regarding dementia prevention programs elicited abstract and hypothetical responses from participants. All interviews were audio-recorded, and field notes were taken. Interview duration ranged from 54 to 81 min. Audio-recordings were transcribed verbatim by a transcription company. Confidentiality was ensured. Transcripts were not returned to participants for correction. Repeat interviews were not performed. Saturation was discussed within the interview team after 12 and 15 interviews. After 15 interviews, no new information was mentioned by participants. Two more interviews were performed to confirm saturation.

### Data analysis

All transcripts were independently coded line-by-line by two researchers (*IV* and *EV*). Consensus meetings were held after each interview. In case of disagreement, a third researcher was consulted (*MP*). First, line-by-line codes were identified by *IV* and *EV*: all text was read and screened line-by-line, and any information deemed relevant to the research question received an initial code. Line-by-line codes (*N* = 2606) were condensed into open codes (*N* = 333) by *IV*. Ninety-one percent of the open codes were identified after inspection of 50% of the line-by-line codes. Open codes were iteratively discussed with *MP*, *RM*, and *MVD*, and further condensed and grouped into 129 code groups. Next, categories were identified by *IV* and iteratively discussed with *MP*, *RM*, and *MVD*, resulting in 21 categories. Out of these categories, five overarching themes were identified. The coding tree of the themes and underlying categories and codes can be found in Supplementary Figures 1–5 (see online supplementary material). Coding was supported by Atlas.ti version 9.

A summary in laymen’s terms was prepared for a synthesized member check. All participants had provided consent to be contacted for a member check. Three participants were not sent the summary [reasons: deceased (*n* = 1), not interested (*n* = 1), nonresponse (*n* = 1)]. The summary was sent to 14 participants, five of whom provided a response. All these participants indicated the themes were clear, accurate, and captured their perspective on social health.

### Research team and reflexivity

All interviews were conducted by *IV* (MD, PhD student at the time of the study), who had previous experience with qualitative data analysis and had undergone training in qualitative research methods. The second coder (*EV*, PhD student at the time of study) had experience in qualitative interviewing and qualitative data analysis. *RM* (MD, PhD, senior researcher), *MVD* (PhD, senior researcher), and *MP* (MD, PhD, senior researcher) were experienced in qualitative research methods. Relationships with the participants prior to the study were not established. Study participants may have had knowledge about the researcher by reading an article on social health in the Rotterdam Study newsletter brief interview with *IV* one year prior to data collection, including limited personal information. The interviewer kept a reflexivity journal to keep track of her assumptions during the interviews. Personal biases and assumptions were discussed during data collection with *MP* and *RM*. Countertransference during the coding process was noted and discussed during consensus discussions between *IV* and *EV*.

## Results

Demographic information of the 17 participants is presented in Table 1. Age ranged from 60 to 74 years. Nine participants were native Dutch and eight participants had an ethnic background from Surinam (*n* = 4), Indonesia (*n* = 3), or Dutch Caribbean (*n* = 1). Many participants self-reported a chronic illness (*n *= 13, 76.5%). Many participants were teachers or had worked in schools in Rotterdam. A slight majority of participants had a higher-intermediate or higher/university level education. All participants lived in Rotterdam, mostly in suburban neighborhoods. The interviews took place between two significant waves of the COVID-19 pandemic, with substantial lockdowns including a recently-lifted curfew.

**Table 1:** Sample characteristics.

#	Age	Sex	Education	Ethnicity	Employment status	Marital status	Lives with housemates	Lonely during COVID-19 pandemic	Chronic disease
**1**	>70	F	Intermediate (higher)	Indonesian	Retired	Widowed	Yes	No	Yes
**2**	>70	M	Intermediate (lower)	Dutch	Retired	Widowed	No	No	No
**3**	<65	F	Intermediate (lower)	Surinamese	Employed	Married/partner	Yes	No	Yes
**4**	<65	M	Intermediate (higher)	Dutch	Sick leave	Married/partner	Yes	Yes	Yes
**5**	65–70	M	Intermediate (lower)	Indonesian	Retired	Married/partner	Yes	Yes	Yes
**6**	>70	M	High/University	Indonesian	Retired	Married/partner	Yes	No	Yes
**7**	65–70	M	Intermediate (lower)	Dutch	Retired	Married/partner	Yes	No	Yes
**8**	>70	F	Primary	Dutch	Retired	Widowed	No	No	Yes
**9**	65–70	F	High/University	Surinamese	Employed	Divorced	No	No	Yes
**10**	<65	F	High/University	Dutch	Employed	Married/partner	Yes	Yes	No
**11**	<65	F	Intermediate (higher)	Dutch	Employed	Never married	No	Yes	No
**12**	<65	M	High/University	Dutch	Employed	Divorced and remarried	No	Yes	No
**13**	65–70	M	High/University	Dutch Caribbean	Retired	Married/partner	Yes	Yes	Yes
**14**	65–70	F	Intermediate (lower)	Surinamese	Incapacitated for work	Married/partner	Yes	Yes	Yes
**15**	65–70	M	Intermediate (lower)	Dutch	Retired	Divorced	Yes	Yes	Yes
**16**	>70	F	Primary	Dutch	Employed	Married/partner	Yes	Yes	Yes
**17**	65–70	M	High/University	Surinamese	Employed	Divorced and remarried	Yes	No	Yes

**Figure 2:**
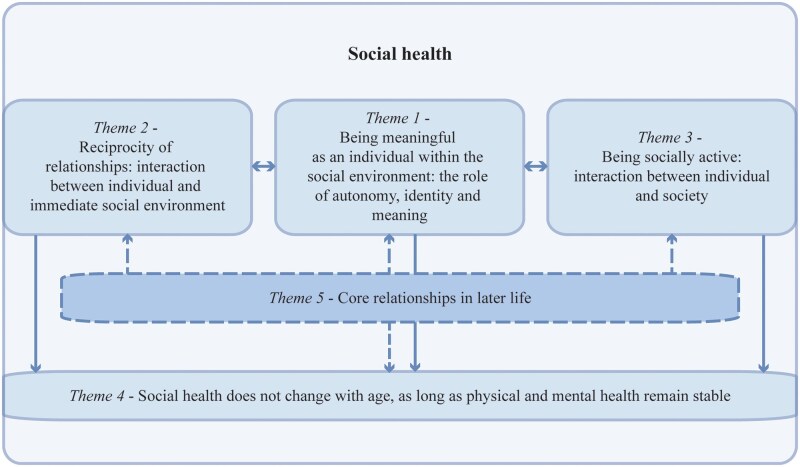
Overview of interconnections between themes.

We identified five themes that capture older adults’ perspectives on the concept of social health (Figure 2). Themes and categories are presented in Table 2. According to the participating older adults, social health is established through interaction between the individual and the social environment on different levels. On the individual level, social health on the first level was being meaningful as an autonomous person within the social environment (Theme 1). In relation to the immediate social environment, social health was having supportive and reciprocated relationships (Theme 2). Within the larger societal context, social health meant being socially active within their needs, while being subjected to social norms (Theme 3). On these different levels of interaction between the individual and the social environment, physical and mental health were preconditions for stable social health in older age (Theme 4), and core relationships with the partner and children were central to all levels of social health (Theme 5). Responsibility for maintaining social health through the process of aging is placed with different parties, corresponding to these different levels of social health. Quotes corresponding to themes and categories are presented in the text and in Table 3. The corresponding code trees per theme are presented in Supplementary Figures 1–5 (see online supplementary material).

**Table 2:** Themes and categories.

Themes	Categories
**Being meaningful as an individual within the social environment: the role of autonomy, identity, and meaning**	Living according to your own norms and autonomy
	Identity shapes social health
	Ways of giving meaning (around retirement age)
	Actions to promote and maintain quality of relationships
**Reciprocity of relationships: interaction between individual and immediate social environment**	Offer and receive support
	(Conditions) values for a mutual relationship
	Experiencing positive interactions between individual and environment: being involved, stimulated, and valued
	Experiencing restrictive interactions between individual and environment: social pressure, rejection, and need to set boundaries
	Dealing with conflict
**Being socially active: interaction between individual and society**	Social structure in which you have social contacts
	Need and pleasure for social contacts
	Perception of meeting (social) norms
	COVID-19 pandemic as litmus test for dealing with social isolation
**Social health does not change with age, as long as physical and mental health remains stable.**	Relationship between social health and overall health
	Changes in social health with age
	Changes in relationships with relatives due to illness
	Dealing with physical limitations and setbacks
	Stigma of getting older and being sick
**Core relationships in later life**	Central role of partner, children, and grandchildren in social health (family that people have founded)
	Importance of maintaining a good relationship with partner
	Relationships outside the family: the role of siblings, in-laws, and friends

**Table 3:** Quotes corresponding to the themes and categories.

Theme	Participant ID	Quote
**1**	17a	But I can imagine that other people, for example let me just for convenience take my brother, well they have no control over that. It happens to them and in that phase they still have to find their way about how they will give substance to social well-being. Their place in society. How do I manage my time? What am I going to do? But yes, I think that’s different for each individual. Basically, I’m already directing that for myself. I already have it in my head how it will all have to go.
	7a	We had a very old neighbor who was I believe 94 who said, I have no one left because I can no longer talk to anyone who has experienced what I have experienced. The book is finished for me.
**2**	9a	Yeah, yeah, you can’t live for yourself, can you? That’s just boring.
	16a	They [family in law] would suddenly drop by. And then—well, I don’t like that. Just call beforehand. Everyone is welcome here. Anyone can come. Everyone always gets something to eat and drink. … I also don’t like it when I don’t have anything in the house, you know when they come over. Just call. Well, that hit a nerve for a lot of them and now they don’t come anymore. … They [family in law] also don’t ask about him [partner] like how is he? Or that you get a phone call. Nothing.
**4**	4b	Well look, those depressions and no work, no income, no money, car is gone, driver’s license gone, your mobility returned to the metro, so to speak. Going to visit someone without a car, using the subway and then it also has to be within a feasible walking distance from the station for me. So that I’ll arrive alive so to speak. Well and that makes your social circle shrink very much.
	17d	Something in my relationship did change for a while during that phase. And by that I mean more of that understanding. Understanding how I feel and how I experience things now despite the fact that she has also experienced it from the beginning, right? But for the partner it stops but for me it didn’t stop. Do you understand? So well, I wouldn’t say the relationship came under tension, but every now and then you kind of felt like you weren’t really being heard.
	13b	My wife doesn’t always have the same amount of pain. … If it gets a little more, it creates a grimmer, not a fun one—I’d give a lot for that to get better. We’ve been together for 40 years now, so I’d really appreciate that. But well, I don’t see that chronic pain going away anytime soon. In my career I could find fantastic solutions for anything and everything. And I can’t find a solution for this. … Then you feel very much in a corner you can’t get out of.
	16d	Well, I think that is a factor that is lacking in social health for us. At least for me. My husband is more satisfied in—He has accepted his fate more. I can’t accept it. And that’s something—You have to deal with it. But accepting, I think that’s a completely different story.
**5**	16e	A lot of my social health depends on his mobility and his well-being. You would say well, but you also have a life yourself and that is true. But you are in one house together. You are married together. You’ve been together for over 50 years. So, you’re not going to say well, just figure it out yourself. I’m going to do my thing. At least not us anyway. Not me. I think there would be—Yes, if there was a little more attention to his mobility, other than physiotherapy twice a week, that someone would offer help once in a while. Try going for a walk with him. Yes, that would help me a lot.
	13c	Well, what I do notice is that—I don’t know if that happens with all people who get older—is that you lose a certain flexibility and suppleness and that you are less willing to make concessions on a number of things. If you—I think if you keep practicing that a little bit so that it stays there, that it affects your contacts with anyone, with your kids, with your wife, with your neighbors or with people you exercise with, that makes it all a lot easier.

### Theme 1. Being meaningful as an individual within the social environment: the role of autonomy, identity and meaning

- Quote 6a: “Social health is also being able to make those choices to use that space that you still have.”
- Quote 6b: “I live my own life. You can think along with me and you can give me advice, but I make my own choices. So in the concept of social health, I attach great importance to freedom of choice in all kinds of areas.”

The freedom to make your own decisions and take your own actions in every part of life were considered essential parts of social health. Autonomy and having meaningful goals were central components of social health on an individual level (Quote-6a, Quote-6b). Goals, meaning, and autonomy were shaped by identity. Identity was often formed in the social context participants grew up in and played a continued role in social health during aging. For some participants this meant that—often negative—childhood experiences affected the decisions they made and make regarding social relationships. For others, cultural identity was more prominent in shaping goals and decision making. Some participants who immigrated to the Netherlands mentioned their considerations for remigration (Quote-3a), cultural norms for social relationships, taking care of one another and keeping in touch with family abroad. Sharing a cultural background or having shared experiences fostered recognition, connecting personal identity to other people (Quote-5a, Table 3-Quote-7a).

- Quote 3a: “Look, if my health permits then I want—Well, not that I really want to. My children are here. But because my husband really wants to [go to Surinam], we can live there for a few months in Surinam and we can come back here [to the Netherlands].”
- Quote 5a: “They [religious community] are important because they are, let’s say, not only fellow believers, but they also feel the same about my teachings, you know? So it feels good. Basically, that they have the same kind of thoughts or the same kind of way of life. That’s pleasant.”

Participants indicated numerous different ways of finding meaning and goals in this life phase. For some participants, meaning was found in work: this could be volunteer work, or continued paid employment after retirement, especially for participants who saw work as a part of their identity. Faith, learning new things, and hobbies were other sources of meaning and goals. By considering autonomy as a central part of social health, they appointed the responsibility for maintaining social health to the individual: one’s own attitude and actions can help investing in social relationships. Participants indicated that personality plays a part in this too: some personality traits (e.g. being passive) would challenge the personal responsibility on social health (Table 3-Quote-17a).

Social health described in this first theme takes place on the level of the individual, incorporating identity, and life’s experiences into making meaning and your own decisions as an older adult. Being an individual person with autonomy and meaning within a larger social environment provided the basis for interactions with this environment in the following themes.

### Theme 2. Reciprocity of relationships: interaction between individual and immediate social environment

- Quote 17b: “It [being there for someone] contributes to my social health in the sense that it just makes me feel really good about it.”

For many participants, social health meant having pleasant contact with others, in which social support was an essential component. In the immediate social environment (i.e. family, friends, neighbors), offering and receiving social support was one of the main ways of interacting. Being there for one another was of immense value, which on the one hand inspired the desire for reciprocated, mutual social support but also opened a gate for disappointment and resentment when the expected support was not received. Being there to support another person was seen as logical, natural and made participants feel good (Quote-15a, Table 3-Quote-9a, Quote-17b).

- Quote 15a: “Yes, it makes sense that you are there for each other. Yes, for me helping other people if that’s possible, yes that is the most normal thing in the world in my opinion. I think I was raised that way.”

Different ways of supporting the immediate social environment were described by the participants: from lending an ear, to driving someone to the hospital without having to be asked, to calling after a medical appointment and asking detailed questions. Financial support was usually only reserved for close family members or friends. Many participants described sharing a meal or providing food as a way of showing support (Quote-14a).

- Quote 14a: “A listening ear—I say maybe I can’t help you with money, but I can always give you a plate of food.”

The desire for reciprocated social support also became apparent when participants spoke of keeping in touch with their immediate social environment. Mutual social relationships could be achieved by meeting specific conditions that are valued aspects of reciprocity. For example, participants disliked being the one who always has to reach out to a specific person in their social environment (Quote-10a).

- Quote 10a: “You know, while my sister is on social media and says hey, do that [join social media] too because then you can also see what I do, you know, then you are involved. Then, I think sure, that’s true, but you could also call, then I’m also involved, you know.”

Overall, positive aspects of interactions were described as being valued, involved and stimulated by the environment. Negative aspects of relationships were described as experiencing social pressure, being rejected, and feeling the need to set boundaries. Moreover, many participants described conflicts with family members for a wide variety of reasons. These conflicts had a profoundly negative effect on emotions and behaviors, were sometimes long-lasting and often unresolved (Table 3-Quote-16a). Participants tried to cope with conflicts in different ways. Although the wish to resolve conflicts was expressed, this approach was often replaced by avoidance or trying to let it go once the feasibility of resolving seemed to have been lost (Quote-13a).

- Quote 13a: “Yes, I’ll just ignore them [the family] then. I try to be as correct as possible and ignore all negativity. [Interviewer: Does that work at that moment?] No, not really. No, it does get under your skin. You’re just trying to do your things, but on the inside that—No, it doesn’t really work no.”

Where social health was described as the individual’s responsibility in Theme 1, maintaining or improving social health here was seen as a responsibility of the immediate social environment: family and friends were considered to be the immediate source of social support. Not only were they the first in line to ask for help, they were also expected to provide support without being asked.

### Theme 3. Being socially active: interaction between individual and society

- Quote 15b: “So I’m not behind the geraniums. Let me put it that way. Some [people] are, of course.”

When asked what social health means to them, many participants used a well-known Dutch expression: social health was not “sitting behind the geraniums,” which roughly translates to “sitting on the sidelines” and is commonly used to describe lonely, older, inactive people. Being socially active and participating was something most participants valued, even if their own need for social interactions was low (Quote-10b).

- Quote 10b: “I love to be with people, but I also have moments where I think oh, wonderful, no one around me, I’m on my own and I’m enjoying myself you know.”

Participants described several aspects of their interaction as an individual with the society as a larger social environment. First, there was the perception of meeting social norms, including, for example, not sitting behind the geraniums in older age. Participants stated that they judged their own and others’ social functioning according to societal norms (Quote-9b, Quote-4a), to feel judged by others, and to avoid negative judgment from the environment by changing their behavior (Quote-5b) by these social norms.

- Quote 9b: “So to indicate in have all kinds of contacts madam. I don’t have to sit alone in my house and waste away. I can always go somewhere.”
- Quote 4a: “You know whether you have a rich and varied social life. And then I think well, then I score very low on that ladder.”
- Quote 5b: “I do try my best, I’d say. I don’t like to pretend to be sick or anything. Just act as normal as possible, I’d say. And that—well, other people don’t need to notice that there’s something wrong with you.”

Another facet of the interaction between individual and society included societal structures that facilitate social contact by providing contexts where people can meet, such as cafes and restaurants, sport clubs, religious communities, and workspaces. In their (sub)urban Rotterdam environment many people live in apartment buildings, where the interaction with neighbors was quite anonymous: neighbors were a part of the larger social structure, not necessarily of the immediate social environment (Quote-8a).

- Quote 8a: “And of course, I also think it’s important how you treat your neighbors. Look, if my neighbors haven’t seen me for a while, I think they’ll alert someone.”

Structures that facilitate social contact were not limited to building structures and organizations, but also included communication means such as mail, telephone, and social media. Participants expressed their preferences for means of communication and indicated that this affected their interactions and well-being (Quote-5c).

- Quote 5c: “I am very good at expressing myself in a letter or an email. Then I can express myself and what I want to say 100%. You know? I always like that. That always gives a little satisfaction.”

Some participants placed the responsibility for social health with the government and with policy, where they specifically mentioned that it was not feasible for them to arrange the care for their health within their immediate social environment. They felt an unrealistic expectation from the government to arrange care within their own social support networks—which they deemed unattainable for older adults with frail networks—rather than the government arranging care for them (Quote-16b).

- Quote 16b: “They’re always talking about that participation society. Well, everyone has their own thing. And everyone that is in your vicinity also has a certain age. You can’t just call on them because the government thinks you should participate and help your neighbors or whatever. I am the youngest of a very large family and the vast majority have already died or are frail.”

The interaction between the individual and society as an essential part of social health was magnified in participants’ experiences of the COVID-19 pandemic: societal changes (e.g. physical distancing measures imposed by governments and changed norms regarding social contact) directly affected individual social health. For many people, this was a confrontation with sudden social isolation: contacts and activities decreased, either because of active avoidance out of risk reduction, or because of losing touch with persons in the social network. Where some were able to compensate for the lack of social activities (Quote-9c), others struggled with being alone and missed their family and friends, even causing physical pain (Quote-3b).

- Quote 9c: “In corona time I would throw some vegetables over the balcony or through the window [of friends’ homes], groceries, because I didn’t dare to go in myself. No, I thought I can’t get corona, you know, and those people have a lot more children and visitors so I’m terrified. Everything happened through the window in that time. It must have looked crazy of course, but that’s how I spent my days.”
- Quote 3b: “Yes, but I know I had a little stomachache but I also felt a bit that the stomachache was not just that. It didn’t come by chance. It’s just more unconscious stress and I know it. […] But yeah, I’m still taking pills for it now.”

### Theme 4. Social health does not change with age, as long as physical and mental health remain stable

- Quote 6c: “So right now I am in pain 24 hours a day. That’s exhausting. That drains mentally. That drains physically. And it also drains socially of course.”

Our participants experienced that social health goes hand in hand with physical and mental health. Several participants experienced that their social environment had shrunk because of their chronic physical and mental health conditions (Quote-6d, Table 3-Quote-4b).

- Quote 6d: “You get all kinds of complaints that make your world smaller. I once described it to the psychologist as in my worst days when I feel myself shrivel up. Then I’m no longer who I used to be. I am no longer the man I used to be. I feel myself getting smaller and smaller. And when you experience that, your whole world becomes smaller.”

Retirement and changing family dynamics with aging further contributed to the feeling that the social environment became smaller. Participants dealing with illness found that it affected their relationships. This mainly had consequences for the relationship with the partner, due to less shared activities and a lack of understanding and acceptance of the disease process by the partner (Quote-17c, Quote-4c, Table 3-Quote-17d).

- Quote 17c: “Yes, you talk to them, but at some point you also start to notice that people understand [my health experience]—She [partner] doesn’t understand. It’s too abstract for her because apparently you can’t convey your feelings or something in a good way.”
- Quote 4c: “When we go out together in the evening to walk the dog, I am holding the dog and not her [partner], because she keeps walking. And then yes, you don’t run after her like a handicapped person. I have to go at my pace you know. And that is sometimes difficult. Also for the other, because they have to dial back.”

When mental and physical health were stable, many participants experienced that their social health remained stable with age, and they did not expect this to change in the future. They acknowledged that growing old surrounded by friends would be preferable, but that life would still be worthwhile with a smaller social network. People who already had to deal with illness, or who were informal caregivers to a sick partner or loved one, took a different view: social health was something they had to actively maintain and work for (Table 3-Quote-13b). Some had already made preparations to maintain their social health in the future, for example, by buying a walker or wheelchair to be able to still visit the museum with their partner in case their physical health deteriorated further.

Participants with a disease (e.g. chronic pain, stroke, heart disease, lung disease, depression) had difficulties coping with their own limitations: they did not want to be limited by pain or mobility issues. Some people tried to avoid certain situations that would adversely affect their health. Some would avoid the topic in conversations to not burden the people around them with their troubles (Quote-15c, Quote-4d).

- Quote 15c: “No, but he tries with humor to take away the misery for himself and for the others too, I think.”
- Quote 4d: “So you’re also teaching yourself [to not open up] because what matters to me right now, what I’d most like to talk about is burdensome for other people—At least, that’s what I think to myself.”

Acceptance was seen as a process, but not unattainable, and would affect social health and social interactions (Table 3-Quote-16d). Conversely, social health also affects physical and mental health, especially in the case of social stress or pressure. Finally, social health was affected not just by overall health of the individual, but crucially also by the health of the partner or other close relatives (Quote-16c). Pain, limited mobility, and depression of either member of a dyad were described to affect relationships (Quote-6c, Quote-3c).

- Quote 16c: “Well, I had imagined something different for my old age. For example, that we could do fun things together and that you are not confined to your home. I think that is part of what negatively affects my social health. That you—Yes, so let’s say you can grow old together nicely. I think that’s social health.”
- Quote 3c: “I mean, when you’re in pain, you’re different. Then you are no longer enjoyable yourself. You want to avoid a lot of things.”

### Theme 5. Core relationships in later life

- Quote 16g: “We are actually quite lonely. Well, not together. Together—We are lonely together.”

The importance of a good relationship with the partner was often mentioned (Quote-16f), although it required time and energy to maintain. Even when the relationship with the partner had changed drastically because of chronic illness, participants tried to maintain the relationship, although this sometimes negatively affected both their own and joint well-being and social health (Quote-16g).

- Quote 16f: “Being able to do things together. Look, luckily I can still go everywhere, but it’s no fun if you have to go without your partner.”

The partner and children were considered core relationships in later life by many participants and as such play a central role in social health: one expression of this was that social health was influenced by the well-being of the partner and children (Table 3-Quote-16e). In the case of divorce, many people tried to stay in touch with their children and ex-partner, even though this added social pressure to the children as one participant recounted (Quote-12a).

- Quote 12a: “While you were already getting divorced from your conflict situation. Already renouncing your social pressure [from family members]. Not realizing that if you leave that it actually contributes [to social pressure for children] even more. You don’t realize that.”

Termination of the relationship or the death of the partner was described as very impactful by most people. Outside of the family that participants had founded (e.g. spouse and children), the family that they grew up in (i.e. parents and siblings) was continuously considered as a source of core relationships. Maintaining their bond with brothers and sisters was important to many participants. Finally, the role of friends was described, often involving long-term friendships. For many people, however, the role of friends was smaller than that of (in-law) family.

This last theme is represented in all the other themes. It shows that, at a later age, core relationships are the key component of perceived social health: these are the people by and for whom identity and meaning-making as a person is formed (Theme 1), with whom mutual relationships are maintained (Theme 2; Table 3-Quote-13c), from whom social norms are derived and imposed on, and with whom contact is maintained within social structures (Theme 3), and who remain central when the social network shrinks and general health imposes restrictions (Theme 4). Figure 2 shows how these themes interact and come together to form social health. Quote-15d illustrates how in one interaction around being a single father, all five themes that together make up social health are intertwined. Quote 15d: “So that is very recognizable to me with taking care of that little one (Theme 1, shared experience, identity). That’s the most important thing in his life (Theme 5, children). … And I understand that. I get it. Yes. And yes, at one point he also played darts, but then again—that was usually on Thursdays. If it was his day [to take care of this daughter], he wouldn’t play darts. Yes, I get that. But his dart mates don’t understand that. ‘You’re just talking about the little one and this and that’ (Theme 3, norms). So yes, you have people like that too. I say yes, dude. You’re just right. I say stop it [playing darts] then (Theme 2, support). Yes, he stopped then (Theme 4, shrinking social environment). Then don’t play darts. The little one is important. Well and that’s how it works.”

## Discussion and implications

In this qualitative study, we identified five themes that capture older adults’ perspectives on the concept of social health. Participants considered their social health to exist on an individual level (Theme 1), in interaction with the immediate social environment (Theme 2), and within the larger societal context (Theme 3). Responsibility for maintaining social health through the aging process was placed with different parties, corresponding to different levels of social health. Social health was experienced as stable, as long as physical and mental health were stable (Theme 4). Core relationships in older age mainly involved family members (Theme 5).

This study’s themes show that social health pervades all aspects of life, social and societal interactions and is deeply intertwined with physical and mental health. This reflects a broader view on social health than Huber’s definition (Figure 1), in which social health is an individual state, albeit affected by external conditions including social and environmental challenges (Huber et al., 2011). Interaction with the social environment and the quality of these interactions was much more emphasized by older adults in our study than the individual balance between opportunities and limitations. The themes in our study demonstrate that to older adults, social health is not merely affected by external social conditions: external social conditions are a crucial component of social health. This also challenges the definition of health by WHO: a state of complete social well-being takes on different meanings for different people in different contexts, and as such is a highly subjective, but societally embedded experience. Recognition of social health as a part of health, interconnected with physical and emotional health, may aid prioritization of social health and relationships in health policy and management. As such, the themes presented here fit more closely to the recently adapted definition of social health, in which social health is proposed to be a reciprocal relational concept involving an individual and their social environment together (Vernooij-Dassen et al., 2022). Together with this adapted definition, a conceptual framework including individual and environmental levels of social health was proposed (Vernooij-Dassen et al., 2022), to which most of the our themes broadly align. The framework’s environmental level focuses on the immediate social network and does not include the societal environment, which was identified by our participants as part of their social health.

Although the current study is the first qualitative study that specifically focused on social health in community-dwelling older adults, other qualitative studies have aimed to answer similar questions on related concepts, such as purpose in life, social support, and peer relationships in older age (AshaRani et al., 2022; Kang et al., 2020; Sta. Maria et al., 2018). In a systematic review on the conceptualization of purpose in life in older adults, social relationships, mattering to others and having meaningful goals and purpose constituted half of the ways in which purpose in life was defined (AshaRani et al., 2022). The aspects of purpose, meaning and mattering to oneself and others correspond to our first theme (individual level social health). Our second theme highlighted the importance of the immediate social environment: social relationships can be greatly supportive, but both their absence and the quality of their presence can have profoundly negative effects on well-being. A qualitative study with Filipino older adults similarly highlighted nuances of support and nonsupport between the individual and people with different amounts of closeness to them: support was characterized as emotional care, instrumental support, companionship, and social connectedness, whereas nonsupport included disrespect and lack of understanding, difficulty in maintaining connections, constraining one’s actions or hindrance in playing an expected/desired role (Sta. Maria et al., 2018). These themes correspond closely to positive and negative aspects that participants mentioned in theme two. A qualitative study among nursing home residents explored social relationships within the nursing home and identified four themes that also closely align with our themes, including how peer relationships promote a sense of belonging; family relationships support a sense of continuity; reciprocity and mutual respect in relationships support a sense of significance; and organizational factors form barriers to maintaining meaningful relationships (Kang et al., 2020). In adding to the existing body of literature on social relationships and well-being in older age, we think it is important to re-iterate that social health encompasses many previously-explored aspects of social relationships, but is distinct in the way that it is a domain of health, rather than a separate entity outside of health. In addition, our social health approach goes beyond well-known and often-applied social markers such as social support, social connectedness, and social networks, and revealed the importance of social aspects on the individual level of autonomy and identity, and the societal level of norms and context.

Perceived social network shrinkage with aging may have far-reaching consequences on well-being (Lang, 2001). Conversely, close emotional ties are relatively stable and continue until late in life (Lang, 2001), which corresponds to our findings in theme four and five: mainly family ties (including the partner) were central to social health. This may indicate that family ties, even when they pose considerable strain, are not typically discontinued, as family ties may be perceived as or expected to be sources of unconditional support, across geographical and cultural variations (Daatland, Herlofson, & Lima, 2011). Loss of core (family) ties may affect perceived social network shrinkage and its consequences more than global network shrinkage including not-close ties: purely measuring network size may therefore not be informative for perceived social network shrinkage.

Our study contributes to a better understanding of social health in older age and identifies aspects of social health that can be improved upon when desired. This has implications for clinical and public health practice, considering older adults’ perspectives on health and successful aging are much broader than the clinical focus on physical health (Domajnko & Pahor, 2015; Teater & Chonody, 2020; Tkatch et al., 2017). Optimizing health in older age should, thus, include optimizing social health, as older adults may be able to compensate for physical health through social health. Healthcare professionals (e.g. primary care, mental health, geriatricians) could play a role in signaling and initiating conversations on future social health changes, upon a physical or mental health challenge. As according to our participants, part of the responsibility for maintaining social health lies with the individual itself, starting conversations on social health may include for older adults to challenge preconceived ideas about aloneness and shrinking social networks from daunting loneliness to opportunities for (positive) solitude (Rokach & Chan, 2021). Education to (older) adults on social health may play a crucial role in this, to help alleviate the current taboo on loneliness and social isolation in older age.

Finally, our study touches on several policy issues that should be addressed in further research. As one participant addressed, families are relied upon to arrange care for their aging family members, in the shortage of paid care workers or the absence of a system that delivers appropriate aging care. This assumes a family structure and health status that is not always present, and places unrealistic expectations on aging couples that may not have the network that is assumed will care for them. Similarly, follow-up research on the interactions between societal changes, policy and individual social health is still desperately needed in the aftermath of the COVID-19 pandemic and in a time of growing societal polarization and individualization. Previous research on loneliness has similarly highlighted implications for policy (Dahlberg et al., 2022; Tong et al., 2021), and we encourage further research that may inform health and aging policies in the realm of social health.

This study has several strengths and limitations. The study sample was ethnically diverse and included perspectives and experiences of people with a variety of (bi-)cultural backgrounds, which allows for a richer perspective on social health. Data saturation was broadly achieved. We applied quality criteria for qualitative research, including investigator triangulation, reporting on reflexivity, inviting a member check. Limitations of our study include that our study process was less iterative than ideally done in qualitative research. Due to COVID-19 physical distancing and lockdown measures during the interview phase, interviews were performed in close proximity to each other, which limited time for in-depth data analysis between interviews. The COVID-19 pandemic further may have colored older adults’ perspective toward social health during the pandemic and lockdowns, rather than a general scope of their social health, which may influence the transferability of our results. We think that our results are valid outside the scope of the pandemic since other qualitative studies align with our findings. In terms of transferability to, for example, rural settings, the (sub)urban context of our participants may be considered. Finally, the concept of social health may have been too abstract for laymen participants to reflect upon. Some participants indeed acknowledged that the term “social health” did not mean anything to them and responded better to terms as “healthy social life” or “healthy social relationships.” This may have been a limitation in the sense that asking about social health did not correspond to the abstraction level of the interviewees, but did contribute to information on how social health is understood by the general public. We minimized this limitation by adapting the interview guide during data collection.

## Conclusion

Social health is considered multidimensional by older adults: it is shaped by life experiences from early childhood to late adulthood that contribute to identity, meaning, and autonomy; it moves with the immediate social environment in how reciprocated interactions are perceived; and it takes places within a societal frame that pervades everyday social interactions with core ties. Physical and mental health of the individual and their loved ones are preconditions for stable social health in older age. Social health is not merely affected by external social conditions: social interactions with core ties are a key component of social health. In promoting health in older age, the social health domain is essential to include, since it is central to all aspects of health, function, and life. Focusing on individuals is not enough to promote social health but should include the interaction with core ties in the older adult’s life.

## Supplementary Material

gnag032_Supplementary_Data

## Data Availability

The Rotterdam Study Personal Registration Data collection is filed with the Erasmus MC Data Protection Officer under registration number EMC1712001. The Rotterdam Study has been entered into the Netherlands National Trial Register (NTR; www.trialregister.nl) and into the WHO International Clinical Trials Registry Platform (ICTRP; https://apps.who.int/trialsearch/) under shared catalogue number NL6645/NTR6831. Because of restrictions based on privacy regulations and informed consent of the participants, data cannot be made freely available in a public repository. Data can be obtained upon request. Requests should be directed toward the management team of the Rotterdam Study (datamanagement.ergo@erasmusmc.nl), which has a protocol for approving data requests. The authors are grateful to the study participants, the staff from the Rotterdam Study and the participating general practitioners and pharmacists.
