# Supplementary material for: “You can’t live for yourself, can you? That’s just boring”: perspectives on social health as a relational concept as described by community-dwelling older adults in a large Dutch city
Source: Gerontologist. 2026 Mar 23;66(5):gnag032. doi: 10.1093/geront/gnag032 (PMC13131960; doi:10.1093/geront/gnag032)
Supplement: gnag032_Supplementary_Data [file gnag032_supplementary_data.pdf]

“You can't live for yourself, can you? That’s just boring” – Perspectives on social health as a relational concept as described by community-dwelling older adults in a large Dutch city

Isabelle F. van der Velpen, MD, PhD, https://orcid.org/0000-0002-0128-6798[1,2], Eline Verspoor, MSc,[3] M. Arfan Ikram, MD, PhD, https://orcid.org/0000-0003-0372-8585[1], Meike W. Vernooij, MD, PhD [1,2], René J.F. Melis, MD, PhD, https://orcid.org/0000-0002-7863-4738[3,4], Myrra J.F. Vernooij-Dassen, PhD [5], Marieke Perry, MD, PhD [3,6]

1. Department of Epidemiology, Erasmus MC, Rotterdam, The Netherlands
2. Department of Radiology and Nuclear Medicine, Erasmus MC, Rotterdam, The Netherlands
3. Department of Geriatric Medicine, Radboudumc Alzheimer Center, Radboud university medical center, Nijmegen, The Netherlands
4. Radboud Institute for Health Sciences, Radboud university medical center, Nijmegen, The Netherlands
5. Department of IQ Healthcare, Radboud university medical center, Nijmegen, The Netherlands
6. Department of Primary and Community Care, Radboud university medical center, Nijmegen, The Netherlands

Corresponding author:
Marieke Perry, MD, PhD, Department of Geriatric Medicine, Radboudumc Alzheimer Center, Radboud University Medical Center. Geert Grooteplein Zuid 10, 6500 HB Nijmegen, The Netherlands. E-mail: Marieke.Perry@radboudumc.nl. Tel: +31 24 36 16 772.

Email addresses
i.vandervelpen@erasmusmc.nl
verspooreline@gmail.com
m.a.ikram@erasmusmc.nl
m.vernooij@erasmusmc.nl
rene.melis@radboudumc.nl
myrra.vernooij-dassen@radboudumc.nl
marieke.perry@radboudumc.nl

Supplemental material

“You can't live for yourself, can you? That’s just boring” – Perspectives on social health as a relational concept as described by community-dwelling older adults in a large Dutch city

Supplemental Table 1. Interview guide

Supplemental Figure 1. Code tree Theme 1

Supplemental Figure 2a. Code tree Theme 2, part 1

Supplemental Figure 2b. Code tree Theme 2, part 2

Supplemental Figure 3a. Code tree Theme 3, part 1

Supplemental Figure 3b. Code tree Theme 3, part 2

Supplemental Figure 4. Code tree Theme 4

Supplemental Figure 5. Code tree Theme 5

Supplemental Table 1. Interview guide

Main topics	Prompts
**Introduction**	
What does an ordinary day look like for you?<br>What does your family and circle of friends/acquaintances look like?	Who are you in contact with? Also by phone or chat.<br>Who are important to you – why?
**Topic 1. What is social health?**	
What do you think of when you think of the term social health?	What is a healthy social life for you?
What do you find important in your social life?	What can others do/mean for you?<br>What can you do/mean for others?<br>Can you cite an example of that?
How do you see your social health within your overall health?	What role does your social life play in how you feel (your well-being)? /How do you notice that?<br>Does your health affect your social life? And vice versa? Are there things you no longer do because of your health problems? How did that happen? What is the role of the people around you in this? Do they make you happy or do they make it difficult for you?
**Topic 2. What is needed to have social health?**	
What can you do yourself to have good social health?	Do you think you can do something yourself to have good contacts? How do you do that?<br>Has your social health changed recently? Did you influence that yourself?
How do the people around you influence whether you have good social health?	Are there people who are currently using energy in your life? Why?<br>Are there people who give you energy? Why?<br>Who would you like to do something with? And why is that?<br>Who would you rather avoid? And why is that?<br>To hinder/encourage: How do they do that?
**Topic 3. How can social health be improved?**	
What do you hope your social health will look like in 5 years?	Why? What do you value about your social health? What do you like less?
What do you need to maintain/achieve your social health in the future?	Whose job is it to improve social health?<br>If you had to give your neighbor a tip about social health, what would it be?
**Closing remarks**	

Supplemental Figure 1. Code tree Theme 1

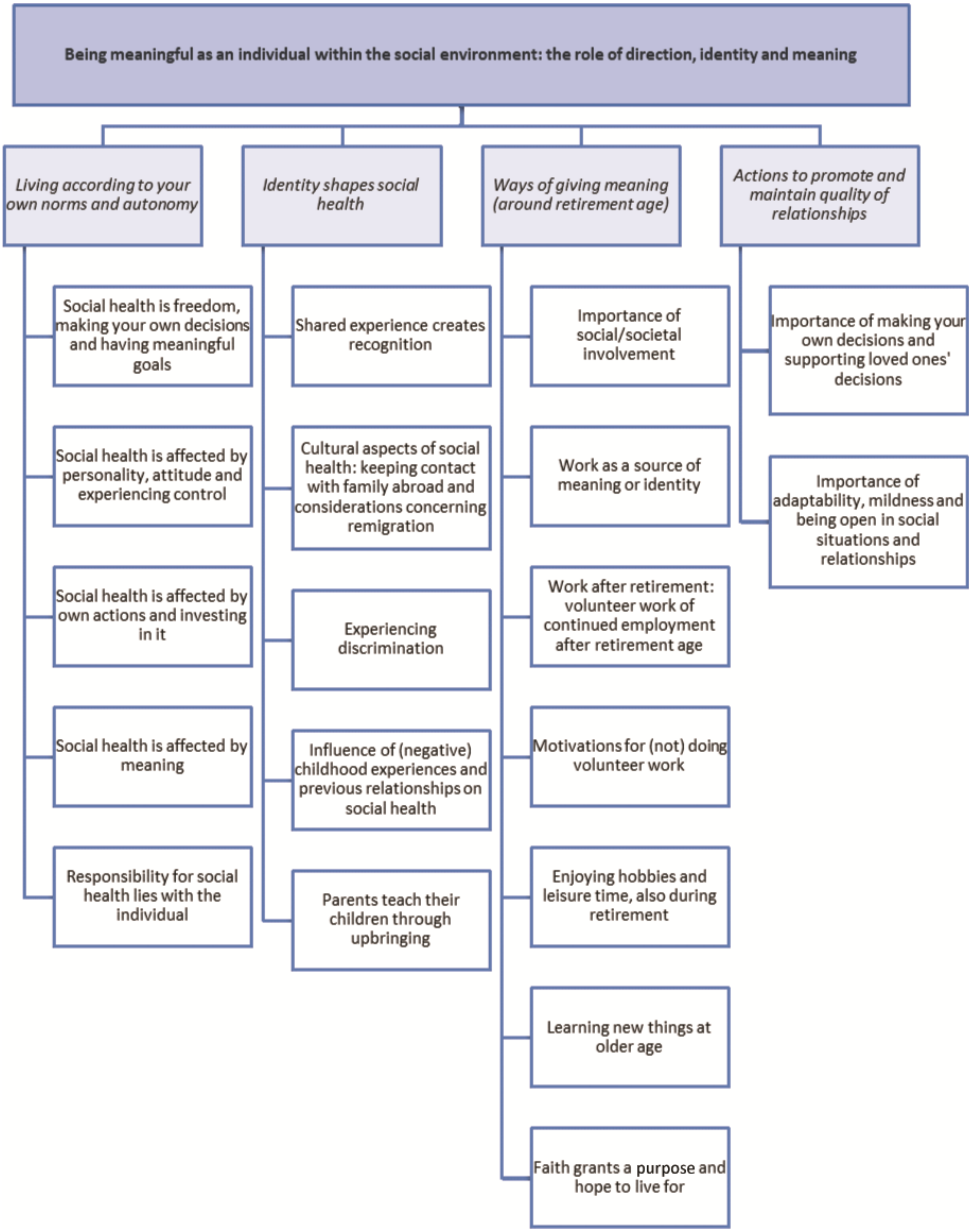

Theme level in dark gray panels, category level in light gray panels, open codes in white panels.

Supplemental Figure 2a. Code tree Theme 2, part 1

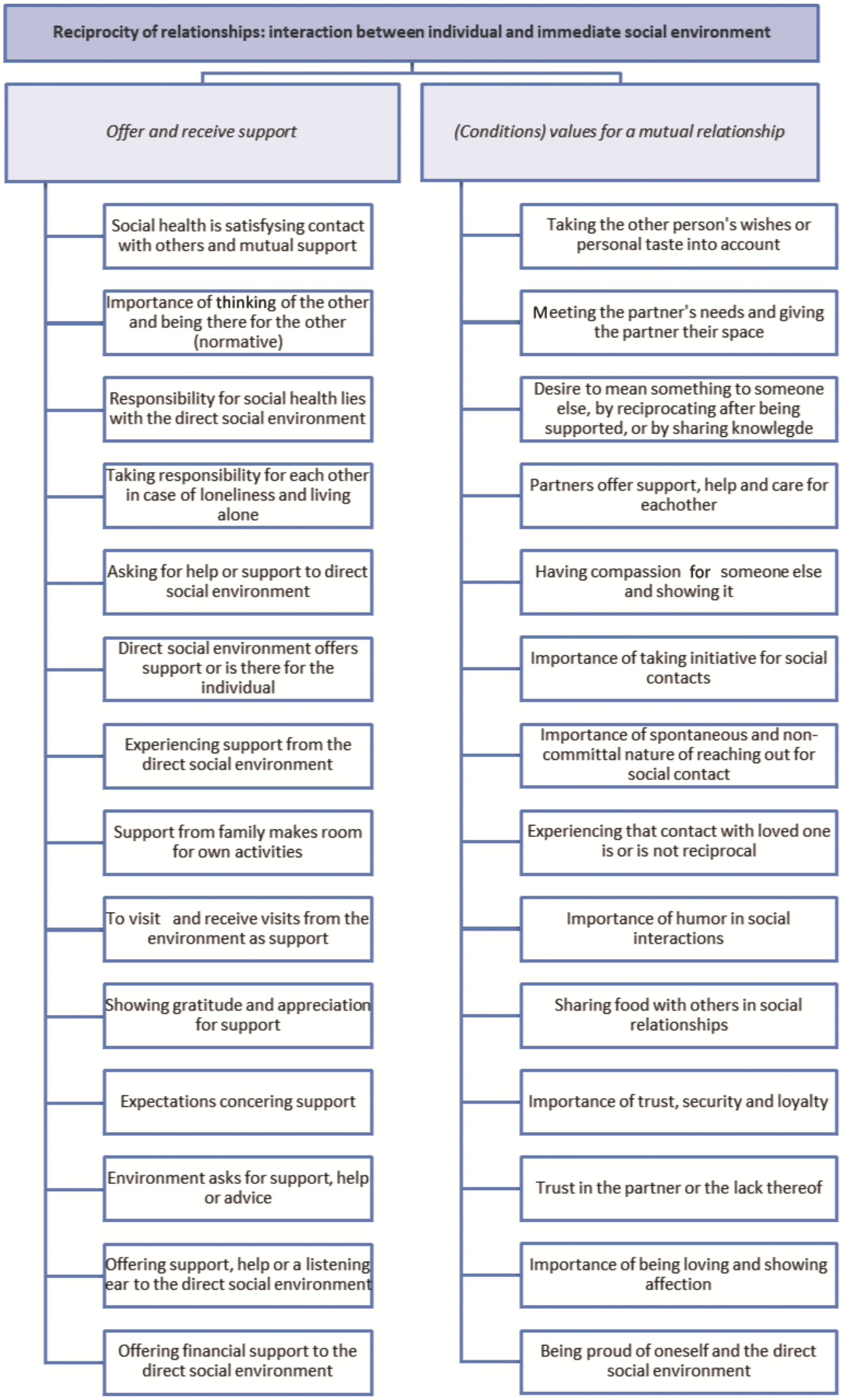

Theme level in dark gray panels, category level in light gray panels, open codes in white panels.

Supplemental Figure 2b. Code tree Theme 2, part 2

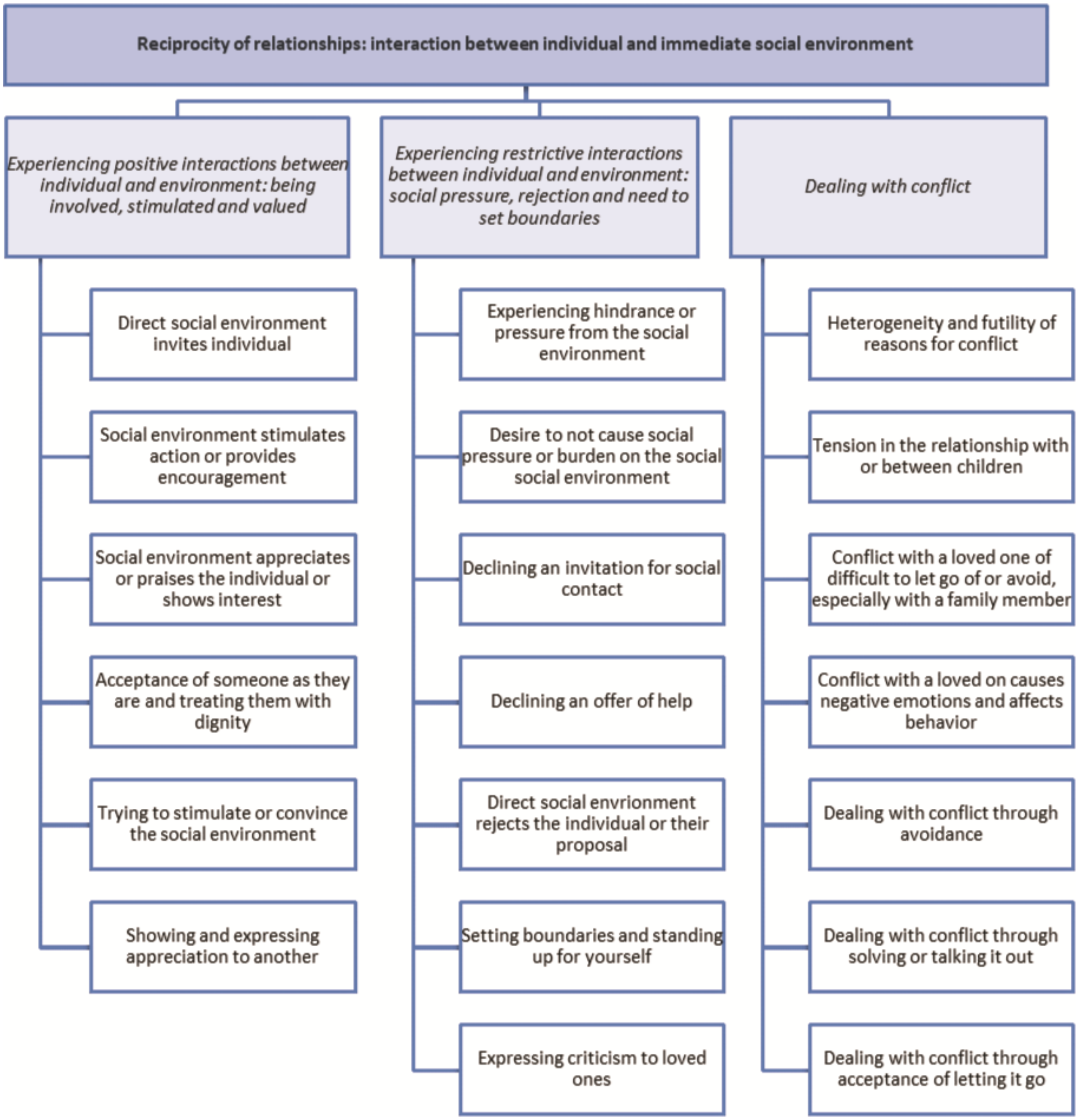

Theme level in dark gray panels, category level in light gray panels, open codes in white panels.

Supplemental Figure 3a. Code tree Theme 3, part 1

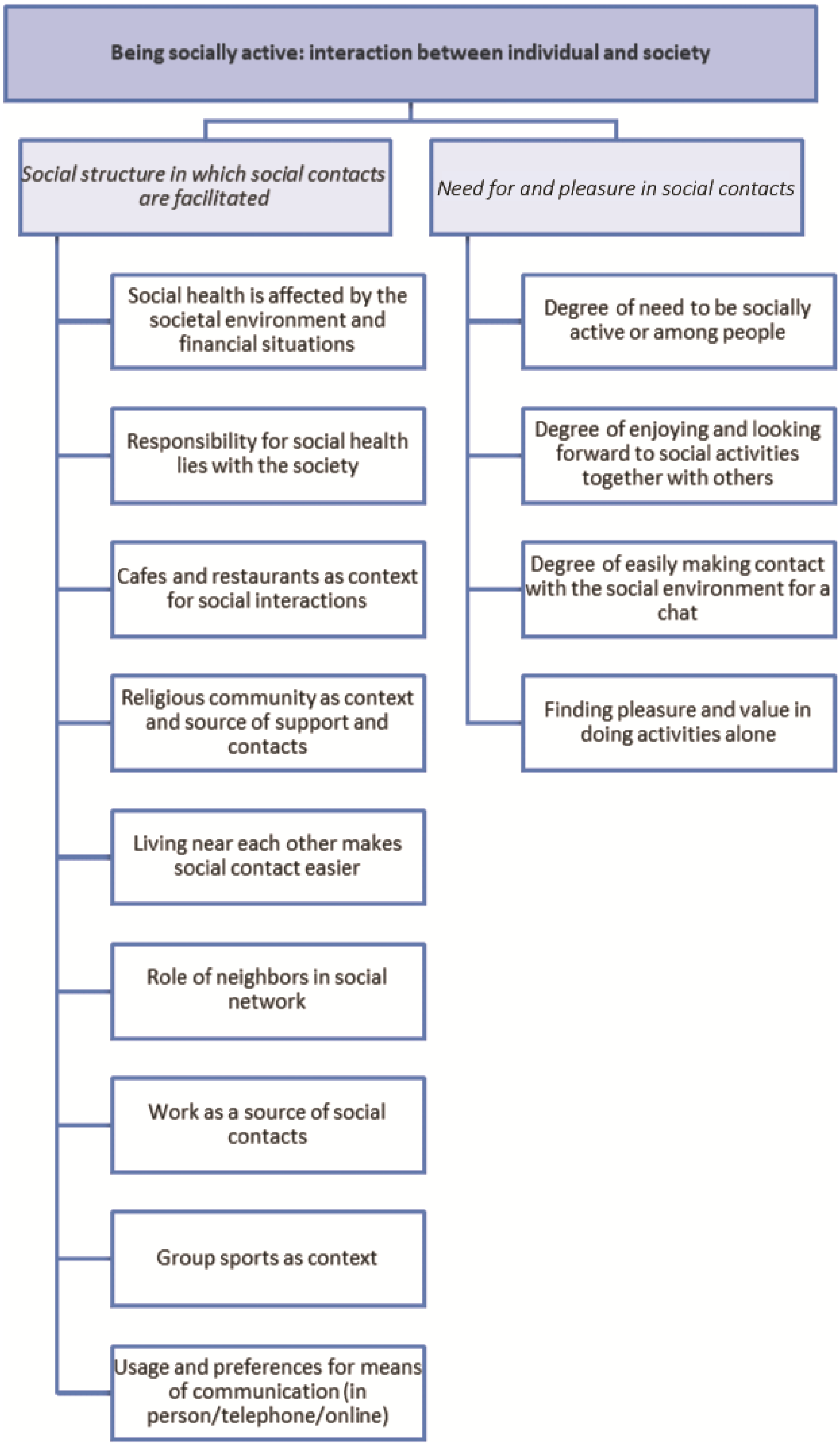

Theme level in dark gray panels, category level in light gray panels, open codes in white panels.

Supplemental Figure 3b. Code tree Theme 3, part 2

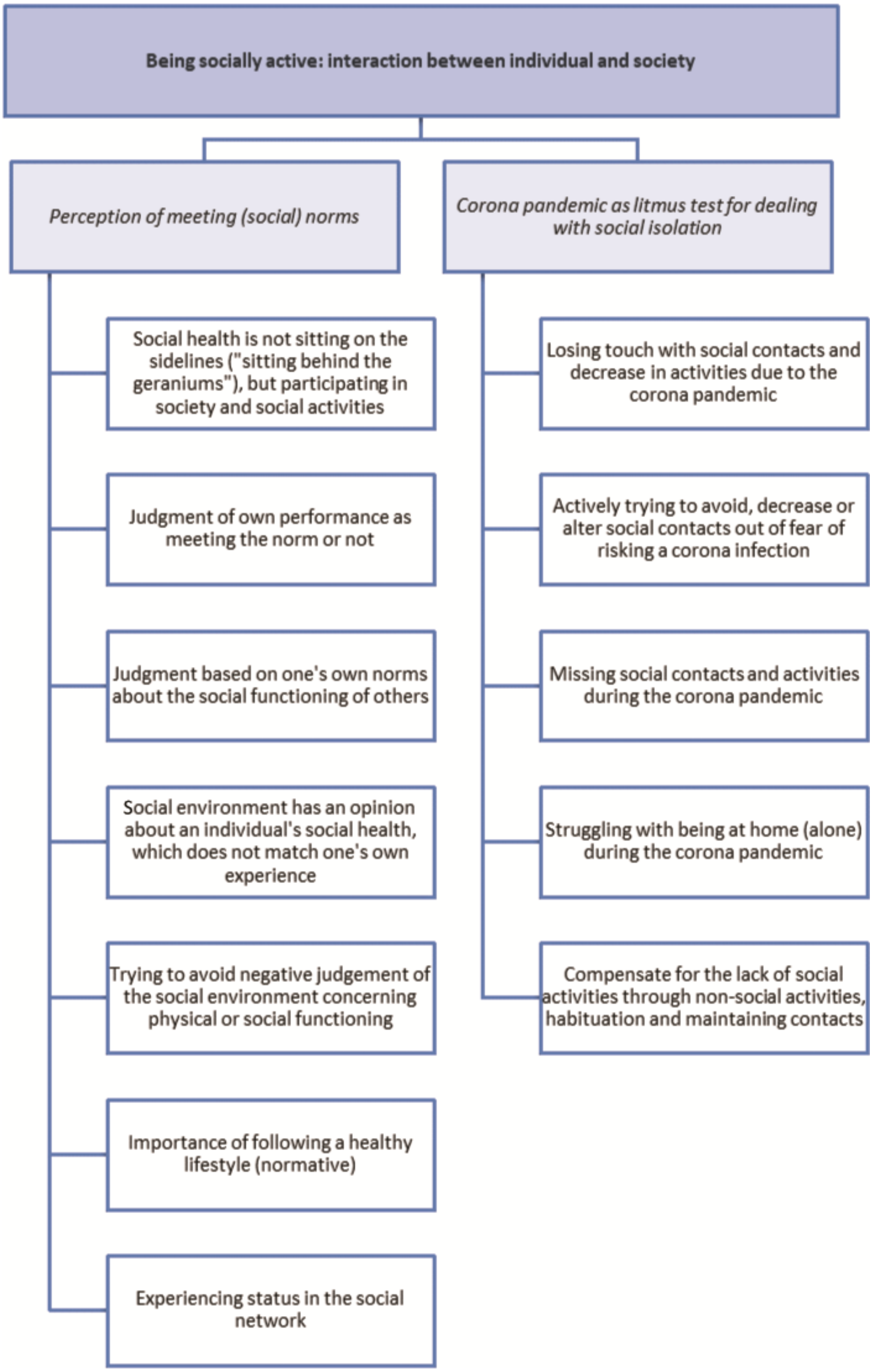

Theme level in dark gray panels, category level in light gray panels, open codes in white panels.

Supplemental Figure 4. Code tree Theme 4

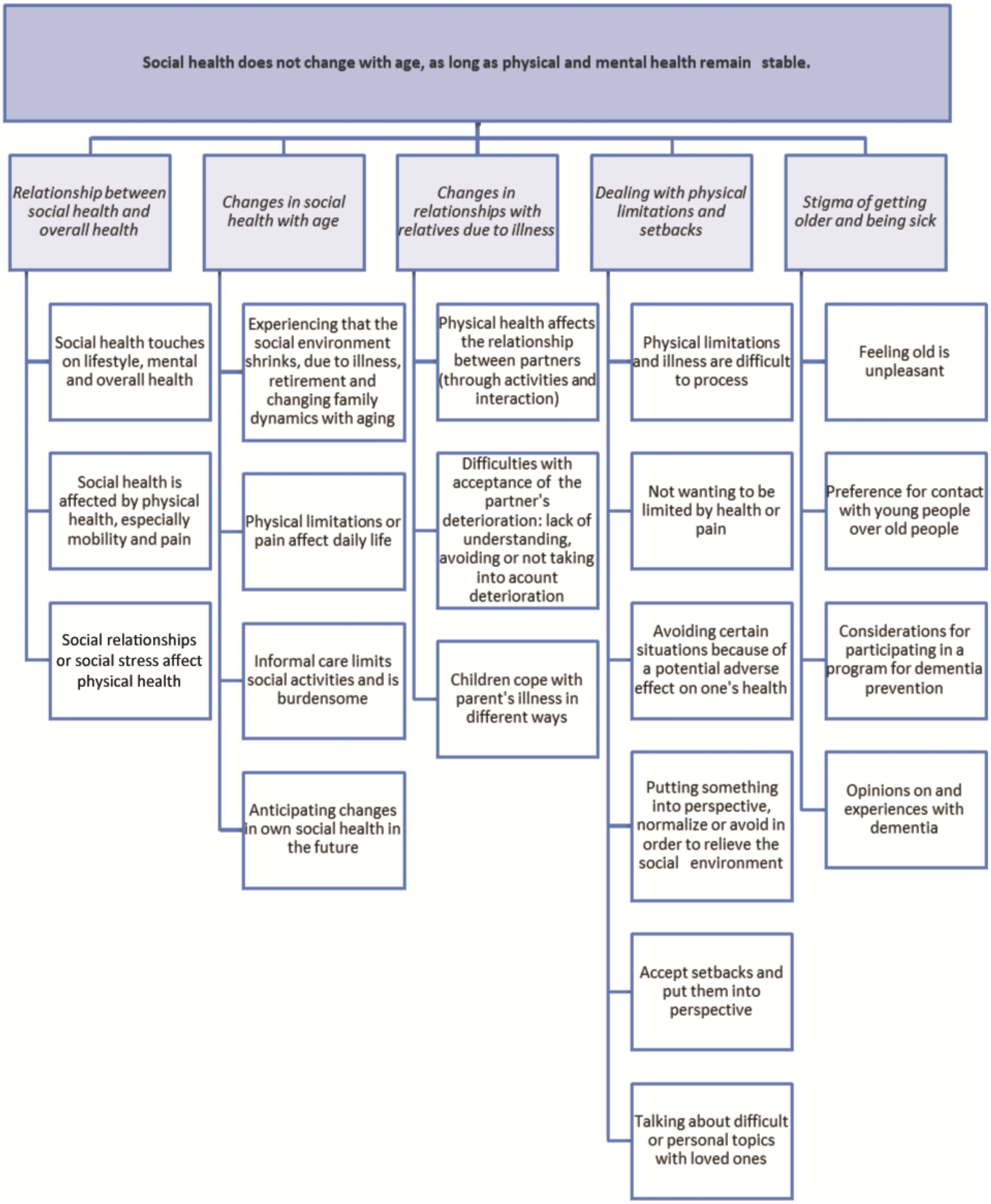

Theme level in dark gray panels, category level in light gray panels, open codes in white panels.

Supplemental Figure 5. Code tree Theme 5

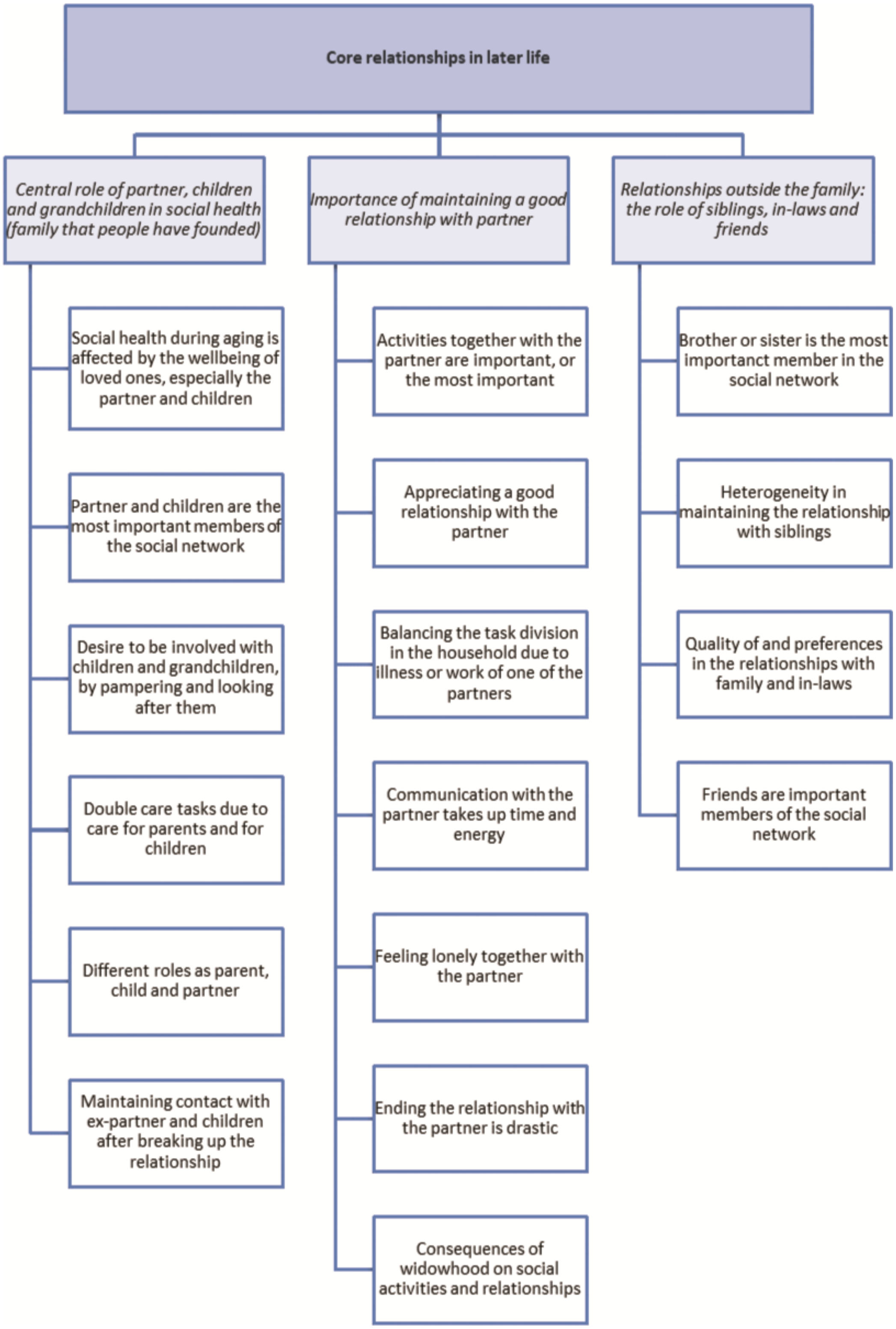

Theme level in dark gray panels, category level in light gray panels, open codes in white panels.
